# Probabilistic Latent Variable Models as Nonnegative Factorizations

**DOI:** 10.1155/2008/947438

**Published:** 2008-05-11

**Authors:** Madhusudana Shashanka, Bhiksha Raj, Paris Smaragdis

**Affiliations:** ^1^Mars Incorporated, 800 High Street, Hackettstown, New Jersy 07840, USA; ^2^Mitsubishi Electric Research Laboratories, 201 Broadway, Cambridge MA 02139, USA; ^3^Adobe Systems Incorporated, 275 Grove Street, Newton MA 02466, USA

## Abstract

This paper presents a family of probabilistic latent variable models that can be used for analysis of nonnegative data. We show that there are strong ties between nonnegative matrix factorization and this family, and provide some straightforward extensions which can help in dealing with shift invariances, higher-order decompositions and sparsity constraints. We argue through these extensions that the use of this approach allows for rapid development of complex statistical models for analyzing nonnegative data.

## 1. Introduction

Techniques to analyze nonnegative data are required in several applications such as analysis of images, text corpora and audio spectra to name a few. A variety of techniques have been proposed for the analysis of such data, such as nonnegative PCA [1], nonnegative ICA [2], nonnegative matrix factorization (NMF) [3], and so on. The goal of all of these techniques is to explain the given nonnegative data as a guaranteed nonnegative linear combination of a set of nonnegative “bases” that represents realistic “building blocks” for the data. Of these, probably the most developed is non-negative matrix factorization, with much recent research devoted to the topic [4–6]. All of these approaches view each data vector as a point in an *N*-dimensional space and attempt to identify the bases that best explain the distribution of the data within this space. For the sake of clarity, we will refer to data that represent vectors in any space as *point* data.

A somewhat related, but separate topic that has garnered much research over the years is the analysis of histograms of multivariate data. Histogram data represent the counts of occurrences of a set of events in a given data set. The aim here is to identify the statistical factors that affect the occurrence of data through the analysis of these counts and appropriate modeling of the distributions underlying them. Such analysis is often required in the analysis of text, behavioral patterns, and so on. A variety of techniques, such as probabilistic latent semantic analysis [7], latent Dirichlet allocation [8], and so on and their derivatives have lately become quite popular. Most, if not all of them, can be related to a class of probabilistic models, known in the behavioral sciences community as *latent class models* [9–11], that attempt to explain the observed histograms as having been drawn from a set of latent classes, each with its own distribution. For clarity, we will refer to histograms and collections of histograms as *histogram* data.

In this paper, we argue that techniques meant for analysis of histogram data can be equally effectively employed for decomposition of nonnegative point data as well, by interpreting the latter as scaled histograms rather than vectors. Specifically, we show that the algorithms used for estimating the parameters of a latent class model are numerically equivalent to the update rules for one form of NMF. We also propose alternate latent variable models for histogram decomposition that are similar to those commonly employed in the analysis of text, to decompose point data and show that these too are identical to the update rules for NMF. We will generically refer to the application of histogram-decomposition techniques to point data as probabilistic decompositions. (This must not be confused with approaches that model the distribution of the set of vectors. In our approach, the vectors themselves are histograms, or, alternately, scaled probability distributions.)

Beyond simple equivalences to NMF, the probabilistic decomposition approach has several advantages, as we explain. Nonnegative PCA/ICA and NMF are primarily intended for matrix-like two-dimensional characterizations of data—the analysis is obtained for matrices that are formed by laying data vectors side-by-side. They do not naturally extend to higher-dimensional tensorial representations, this has been often accomplished by implicit unwrapping the tensors into a matrix. However, the probabilistic decomposition naturally extends from matrices to tensors of arbitrary dimensions.

It is often desired to control the form or structure of the learned bases and their projections. Since the procedure for learning the bases that represent the data is statistical, probabilistic decomposition affords control over the form of the learned bases through the imposition of a priori probabilities, as we will show. Constraints such as sparsity can also be incorporated through these priors.

We also describe extensions to the basic probabilistic decomposition framework that permits shift invariance along one or more of the dimensions (of the data tensor) that can abstract convolutively combined bases from the data.

The rest of the paper is organized as follows. Since, the probabilistic decomposition approach we promote in this paper is most analogous to nonnegative matrix factorization (NMF) among all techniques that analyze nonnegative point data, we begin with a brief discussion of NMF. We present the family of latent variable models in Section 3 that we will employ for probabilistic decompositions. We present tensor generalizations in Section 4.1 and convolutive factorizations in Section 4.2. In Section 4.3, we discuss extensions such as incorporation of sparsity and in Section 4.4, we present aspects of geometric interpretation of these decompositions.

## 2. Nonnegative Matrix Factorization

Nonnegative matrix factorization was introduced by [3] to find nonnegative parts-based representation of data. Given an *M* × *N* matrix **V**, where each column corresponds to a data vector, NMF approximates it as a product of nonnegative matrices **W** and **H**, that is, **V** ≈ **WH**, where **W** is an *M* × *K* matrix and **H** is a *K* × *N* matrix. The above approximation can be written column by column as **v**_*n*_ ≈ **W** **h**_*n*_, where **v**_*n*_ and **h**_*n*_ are the *n*th columns of **V** and **H**, respectively. In other words, each data vector **v**_*n*_ is approximated by a linear combination of the columns of **W**, weighted by the entries of **h**_*n*_. The columns of **W** can be thought of as *basis vectors* that, when combined with appropriate *mixture weights* (entries of the columns of **H**), provide a linear approximation of **V**.

The optimal choice of matrices **W** and **H** are defined by those nonnegative matrices that minimize the reconstruction error between **V** and **WH**. Different error functions have been proposed which lead to different update rules (e.g., [3, 12]). Shown below are multiplicative update rules derived by [3] using an error measure similar to the Kullback-Leibler divergence:

(1) $$\begin{array}{ll} W_{mk} & ⟵W_{mk}\underset{n}{\sum }\frac{V_{mn}}{{\left(WH\right)}_{mn}}H_{kn},\\ W_{mk} & ⟵\frac{W_{mk}}{\sum_{m}W_{mk}},\\ H_{kn} & ⟵H_{kn}\underset{m}{\sum }W_{mk}\frac{V_{mn}}{{\left(WH\right)}_{mn}}, \end{array}$$

where *A*_*ij*_ represents the value at *i*th row and the *j*th column of matrix **A**.

## 3. Latent Variable Models

In its simplest form, NMF expresses an *M* × *N* data matrix **V** as the product of non-negative matrices **W** and **H**. The idea is to express the data vectors (columns of **V**) as a combination of a set of *basis components* or *latent factors* (columns of **W**). Below, we show that a class of probabilistic models employing latent variables, known in the field of social and behavioral sciences as *latent class models* (e.g., [9, 11, 13]), is equivalent to NMF.

Let us represent the two dimensions of the matrix **V** by *x*_1_ and *x*_2_, respectively. We can consider the nonnegative entries *V*_*x*_1_*x*_2__ as having been generated by an underlying probability distribution *P*(*x*_1_, *x*_2_). Variables *x*_1_ and *x*_2_ are multinomial random variables, where *x*_1_ can take one out of a set of *M* values in a given draw and *x*_2_ can take one out of a set of *N* values in a given draw. In other words, one can model *V*_*mn*_, the entry in row *m* and column *n*, as the number of times features *x*_1_ = *m* and *x*_2_ = *n* were picked in a set of repeated draws from the distribution *P*(*x*_1_, *x*_2_). Unlike NMF which tries to characterize the observed data directly, latent class models characterize the underlying distribution *P*(*x*_1_, *x*_2_). This subtle difference of interpretation preserves all the advantages of NMF, while overcoming some of its limitations by providing a framework that is easy to generalize, extend, and interpret.

There are two ways of modeling *P*(*x*_1_, *x*_2_) and we consider them separately below.

### 3.1. Symmetric Factorization

Latent class models enable one to attribute the observations as being due to hidden or latent factors. The main characteristic of these models is conditional independence—multivariate data are modeled as belonging to latent classes such that the random variables within a latent class are independent of one another. The model expresses a multivariate distribution such as *P*(*x*_1_, *x*_2_) as a mixture where each component of the mixture is a product of one-dimensional marginal distributions. In the case of two dimensional data such as **V**, the model can be written mathematically as

(2) $$P(x_{1},\;x_{2})=\underset{z\in \{1,2,\ldots ,K\}}{\sum }P(z)P(x_{1}\;|\;z)P(x_{2}\;|\;z).$$

In (2), *z* is a latent variable that indexes the hidden components and takes values from the set {1,…, *K*}. This equation assumes the *principle of local independence*, whereby the latent variable *z* renders the observed variables *x*_1_ and *x*_2_ independent. This model was presented independently as *probabilistic latent component analysis* (PLCA) by [14]. The aim of the model is to characterize the distribution underlying the data as shown above by learning the parameters so that hidden structure present in the data becomes explicit.

The model can be expressed as a matrix factorization. Representing the parameters *P*(*x*_1_ | *z*), *P*(*x*_2_ | *z*), and *P*(*z*) as entries of matrices **W**, **G**, and **S**, respectively, where

**Figure 1:**
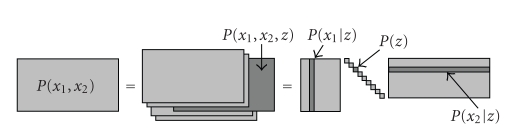
Latent variable model of (2) as matrix factorization.

- **W** is a *M* × *K* matrix such that *W*_*mk*_ corresponds to the probability *P*(*x*_1_ = *m* | *z* = *k*);
- **G** is a *K* × *N* matrix such that *G*_*kn*_ corresponds to the probability *P*(*x*_2_ = *n* | *z* = *k*); and
- **S** is a *K* × *K* diagonal matrix such that *S*_*kk*_ corresponds to the probability *P*(*z* = *k*);

one can write the model of (2) in matrix form as

(3) $$\begin{array}{cc} P & =WSG,\;\text{or equivalently},\\ P & =WH, \end{array}$$

where the entries of matrix **P** correspond to *P*(*x*_1_, *x*_2_) and **H** = **SG**. Figure 1 illustrates the model schematically.

Parameters can be estimated using EM algorithm. The update equations for the parameters can be written as

(4) $$\begin{array}{cc} P(z\;|\;x_{1},\;x_{2}) & =\frac{P(z)P(x_{1}\;|\;z)P(x_{2}\;|\;z)}{\sum_{z}\;P(z)P(x_{1}\;|\;z)P(x_{2}\;|\;z)},\\ P(x_{i}\;|\;z) & =\frac{\sum_{j\in \{1,2\},j\neq i}V_{x_{1}x_{2}}P(z\;|\;x_{1},\;x_{2})}{\sum_{x_{1},x_{2}}V_{x_{1}x_{2}}P(z\;|\;x_{1},\;x_{2})},\\ P(z) & =\frac{\sum_{x_{1},x_{2}}V_{x_{1}x_{2}}P(z\;|\;x_{1},\;x_{2})}{\sum_{z,x_{1},x_{2}}V_{x_{1}x_{2}}P(z\;|\;x_{1},\;x_{2})}. \end{array}$$

Writing the above update equations in matrix form using **W** and **H** from (3), we obtain

(5) $$\begin{array}{c} W_{mk}⟵W_{mk}\underset{n}{\sum }\frac{V_{mn}}{{\left(WH\right)}_{mn}}H_{kn},\;W_{mk}⟵\frac{W_{mk}}{\sum_{m}W_{mk}},\\ H_{kn}⟵H_{kn}\underset{m}{\sum }W_{mk}\frac{V_{mn}}{{\left(WH\right)}_{mn}},\;\;H_{kn}⟵\frac{H_{kn}}{\sum_{k,n}H_{kn}}. \end{array}\;$$

The above equations are identical to the NMF update equations of (1) upto a scaling factor in **H**. This is due to the fact that the probabilistic model decomposes **P** which is equivalent to a normalized version of the data **V**. Reference [14] presents detailed derivation of the update algorithms and comparison with NMF update equations. This model has been used in analyzing image and audio data among other applications (e.g., [14–16]).

### 3.2. Asymmetric Factorization

The latent class model of (2) considers each dimension symmetrically for factorization. The two dimensional distribution *P*(*x*_1_, *x*_2_) is expressed as a mixture of two-dimensional latent factors where each factor is a product of one-dimensional marginal distributions. Now, consider the following factorization of *P*(*x*_1_, *x*_2_):

(6) $$\begin{array}{cc} P(x_{1},\;x_{2}) & =P(x_{i})P(x_{j}\;|\;x_{i}),\\ P(x_{j}\;|\;x_{i}) & =\underset{z}{\sum }P(x_{j}\;|\;z)P(z\;|\;x_{i}), \end{array}$$

where *i*, *j* ∈ {1, 2}, *i* ≠ *j* and *z* is a latent variable. This version of the model with asymmetric factorization is popularly known as *probabilistic latent semantic analysis* (PLSA) in the topic-modeling literature [7].

**Figure 2:**
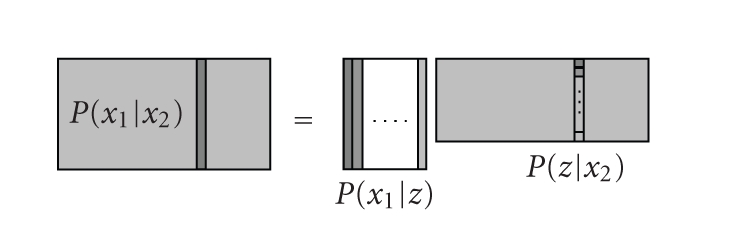
Latent variable model of (6) as matrix factorization.

Without loss of generality, let *j* = 1 and *i* = 2. We can write the above model in matrix form as **q**_*n*_ = **W** **g**_*n*_, where **q**_*n*_ is a column vector indicating *P*(*x*_1_ | *x*_2_), **g**_*n*_ is a column vector indicating *P*(*z* | *x*_2_), and **W** is a matrix with the (*m*, *k*)th element corresponding to *P*(*x*_1_ = *m* | *z* = *k*). If *z* takes *K* values, **W** is a *M* × *K* matrix. Concatenating all column vectors **q**_*n*_ and **g**_*n*_ as matrices **Q** and **G**, respectively, one can write the model as

(7) $$\begin{array}{cc} Q & =WG,\;\text{or equivalently}\\ V & =WGS=WH, \end{array}$$

where **S** is a *N* × *N* diagonal matrix whose *n*th diagonal element is the sum of the entries of **v**_*n*_ (the *n*th column of ** V**), and **H** = **GS**. Figure 2 provides a schematic illustration of the model.

Given data matrix **V**, parameters *P*(*x*_1_ | *z*) and *P*(*z* | *x*_2_) are estimated by iterations of equations derived using the EM algorithm:

(8) $$\begin{array}{cc} P(z\;|\;x_{1},\;x_{2}) & =\frac{P(z\;|\;x_{2})P(x_{1}\;|\;z)}{\sum_{z}\;P(z\;|\;x_{2})P(x_{1}\;|\;z)},\\ P(x_{1}\;|\;z) & =\frac{\sum_{x_{2}}V_{x_{1}x_{2}}P(z\;|\;x_{1},\;x_{2})}{\sum_{x_{1},x_{2}}V_{x_{1}x_{2}}P(z\;|\;x_{1},\;x_{2})},\\ P(z\;|\;x_{2}) & =\frac{\sum_{x_{1}}V_{x_{1}x_{2}}P(z\;|\;x_{1},\;x_{2})}{\sum_{x_{1}}V_{x_{1}x_{2}}}. \end{array}$$

Writing the above equations in matrix form using **W** and **H** from (7), we obtain

(9) $$\begin{array}{cc} W_{mk} & ⟵W_{mk}\underset{n}{\sum }\frac{V_{mn}}{{\left(WH\right)}_{mn}}H_{kn},\\ W_{mk} & ⟵\frac{W_{mk}}{\sum_{m}W_{mk}},\\ H_{kn} & ⟵H_{kn}\underset{m}{\sum }W_{mk}\frac{V_{mn}}{{\left(WH\right)}_{mn}}. \end{array}$$

The above set of equations is exactly identical to the NMF update equations of (1). See [17, 18] for detailed derivation of the update equations. The equivalence between NMF and PLSA has also been pointed out by [19]. The model has been used for the analysis of audio spectra (e.g., [20]), images (e.g., [17, 21]), and text corpora (e.g., [7]).

## 4. Model Extensions

The popularity of NMF comes mainly from its empirical success in finding “useful components” from the data. As pointed out by several researchers, NMF has certain important limitations despite the success. We have presented probabilistic models that are numerically closely related to or identical to one of the widely used NMF update algorithms. Despite the numerical equivalence, the methodological difference in approaches is important. In this section, we outline some advantages of using this alternate probabilistic view of NMF.

The first and most straightforward implication of using a probabilistic approach is that it provides a theoretical basis for the technique. And more importantly, the probabilistic underpinning enables one to utilize all the tools and machinery of statistical inference for estimation. This is crucial for extensions and generalizations of the method. Beyond these obvious advantages, below we discuss some specific examples where utilizing this approach is more useful.

### 4.1. Tensorial Factorization

NMF was introduced to analyze two-dimensional data. However, there are several domains with nonnegative multidimensional data where a multidimensional correlate of NMF could be very useful. This problem has been termed as nonnegative tensor factorization (NTF). Several extensions of NMF have been proposed to handle multi-dimensional data (e.g., [4–6, 22]). Typically, these methods flatten the tensor into a matrix representation and proceed further with analysis. Conceptually, NTF is a natural generalization of NMF, but the estimation algorithms for learning the parameters, however, do not lend themselves to extensions easily. Several issues contribute to this difficulty. We do not present the reasons here due to lack of space but a detailed discussion can be found in [6].

Now, consider the symmetric factorization case of the latent variable model presented in Section 3.1. This model is naturally suited for generalizations to multiple dimensions. In its general form, the model expresses a *K*-dimensional distribution as a mixture, where each *K*-dimensional component of the mixture is a product of one-dimensional marginal distributions. Mathematically, it can be written as

(10) $$P(x)=\underset{z}{\sum }P(z)\overset{K}{\underset{j=1}{\prod }}P(x_{j}\;|\;z),$$

where *P*(**x**) is a *K*-dimensional distribution of the random variable **x** = *x*_1_, *x*_2_,…, *x*_*K*_. *z* is the latent variable indexing the mixture components and *P*(*x*_*j*_ | *z*) are one-dimensional marginal distributions. Parameters are estimated by iterations of equations derived using the EM algorithm and they are

(11) $$\begin{array}{cc} R(x,\;z) & =\frac{P(z)\prod_{j=1}^{N}P(x_{j}\;|\;z)}{\sum_{z^{\prime }}\;P(z^{\prime })\prod_{j=1}^{N}P(x_{j}\;|\;z^{\prime })},\\ P(z) & =\underset{j}{\sum }\;\underset{xj}{\sum }P(x)R(x,\;z),\\ P(x_{j}\;|\;z) & =\frac{\sum_{i:i\neq j}\sum_{x_{i}}P(x)R(x,\;z)}{P(z)}. \end{array}$$

In the two-dimensional case, the update equations reduce to (4).

**Figure 3:**
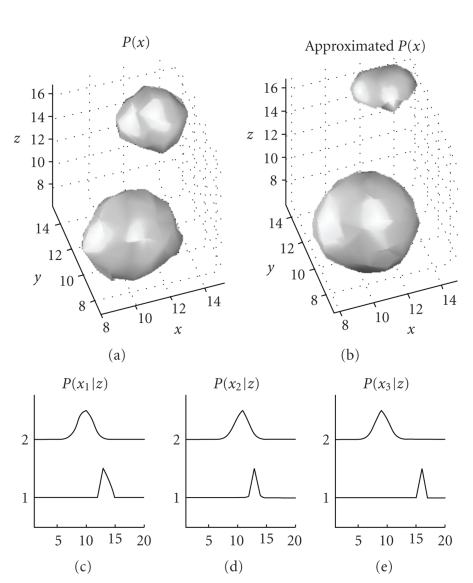
An example of a higher dimensional positive data decomposition. An isosurface of the original input is shown at the top left, the approximation by the model in (10) is shown in the top right, and the extracted marginals (or factors) are shown in the lower plots.

To illustrate the kind of output of this algorithm, consider the following toy example. The input *P*(**x**) was the 3-dimensional distribution shown in the upper left plot in Figure 3. This distribution can also be seen as a rank 3 positive tensor. It is clearly composed out of two components, each being an isotropic Gaussian with means at *μ*_1_ = 11, 11, 9 and *μ*_2_ = 14, 14, 16 and variances *σ* ^2^_1_ = 1 and *σ* ^2^_2_ = 1/2, respectively. The bottom row of plots shows the derived sets of *P*(*x*_*j*_ | *z*) using the estimation procedure we just described. We can see that each of them is composed out of a Gaussian at the expected position and with the expected variance. The approximated *P*(**x**) using this mode is shown in the top right. Other examples of applications on more complex data and a detailed derivation of the algorithm can be found in [14, 23].

### 4.2. Convolutive Decompositions

Given a two-dimensional dataset, NMF finds hidden structure along one dimension (columnwise) that is characteristic to the entire dataset. Consider a scenario where there is localized structure present along both dimensions (rows and columns) that has to be extracted from the data. An example dataset would be an acoustic spectrogram of human speech which has structure along both frequency and time. Traditional NMF is unable to find structure across both dimensions and several extensions have been proposed to handle such datasets (e.g., [24, 25]).

The latent variable model can be extended for such datasets and the parameter estimation still follows a simple EM algorithm based on the principle of maximum likelihood. The model, known as a *shift invariant* version of PLCA, can be mathematically written as [23]

(12) $$P(x)=\underset{z}{\sum }(P(z)\int P(w,\;\tau \;|\;z)P(h-\tau \;|\;z)d\tau ),$$

where the *kernel distribution* *P*(**w**,**τ** | *z*) = 0, ∀ **τ** ∉ *ℛ* where *ℛ* defines a local convex region along the dimensions of **x**. Similar to the simple model of (2), the model expresses *P*(**x**) as a mixture of latent components. But instead of each component being a simple product of one-dimensional distributions, the components are convolutions between a multidimensional “kernel distribution” and a multidimensional “impulse distribution”. The update equations for the parameters are

(13) $$\begin{array}{cc} R(x,\;\tau ,\;z) & =\frac{P(z)P(w,\;\tau \;|\;z)P(h-\tau \;|\;z)}{\sum_{z^{\prime }}P(z^{\prime })\int P(w,\;\tau^{\prime }\;|\;z^{\prime })P(h-\tau^{\prime }\;|\;z^{\prime })d\tau^{\prime }},\\ P(z) & =\int R(x,\;z)dx,\\ P(w,\;\tau \;|\;z) & =\frac{\int P(x)R(x,\;\tau ,\;z)dh}{P(z)},\\ P(h\;|\;z) & =\frac{\int P(w,\;h+\tau )R(w,\;h+\tau ,\;\tau ,\;z)dw\;d\tau }{\int P(w,\;h^{\prime }+\tau )R(w,\;h^{\prime }+\tau ,\;\tau ,\;z)dh^{\prime }dw\;d\tau .} \end{array}$$

**Figure 4:**
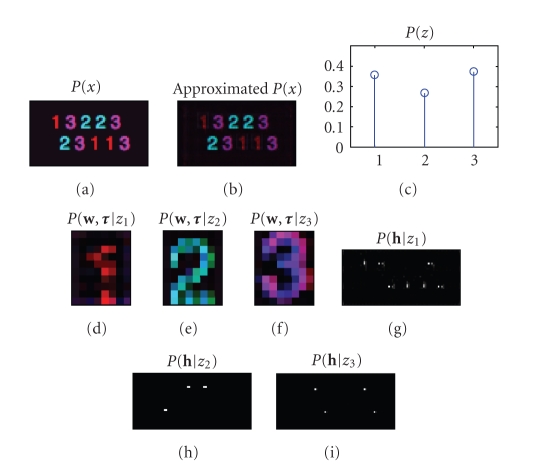
An example of a higher dimensional shift-invariant positive data decomposition. The original input is shown at the top left, the approximation by the model in (12) is shown in the top middle, and the extracted kernels and impulses are shown in the lower plots.

Detailed derivation of the algorithm can be found in [14]. The above model is able to deal with tensorial data just as well as matrix data. To illustrate this model, consider the picture in the top left of Figure 4. This particular image is a rank-3 tensor (*x*, *y*, color). We wish to discover the underlying components that make up this image. The components are the digits 1, 2, 3 and appear in various spatial locations, thereby necessitating a “shift-invariant” approach. Using the aforementioned algorithm, we obtain the results shown in Figure 4. Other examples of such decompositions on more complex data are shown in [23].

The example above illustrates shift invariance, but it is conceivable that “components” that form the input might occur with transformations such as rotations and/or scaling in addition to translations (shifts). It is possible to extend this model to incorporate invariance to such transformations. The derivation follows naturally from the approach outlined above, but we omit further discussion here due to space constraints.

### 4.3. Extensions in the Form of Priors

One of the more apparent limitations of NMF is related to the quality of components that are extracted. Researchers have pointed out that NMF, as introduced by Lee and Seung, does not have an explicit way to control the “sparsity” of the desired components [26]. In fact, the inability to impose sparsity is just a specific example of a more general limitation. NMF does not provide a way to impose known or hypothesized structure about the data during estimation.

To elaborate, let us consider the example of sparsity. Several extensions have been proposed to NMF to incorporate sparsity (e.g., [26–28]). The general idea in these methods is to impose a cost function during estimation that incorporates an additional constraint that quantifies the sparsity of the obtained factors. While sparsity is usually specified as the *L*0 norm of the derived factors [29], the actual constraints used consider an *L*1 norm, since the *L*0 norm is not amenable to optimization within a procedure that primarily attempts to minimize the *L*2 norm of the error between the original data and the approximation given by the estimated factors. In the probabilistic formulation, the relationship of the sparsity constraint to the actual objective function optimized is more direct. We characterize sparsity through the entropy of the derived factors, as originally specified in [30]. A sparse code is defined as a set of basis vectors such that any given data point can be largely explained by only a few bases from the set, such that the required contribution of the rest of the bases to the data point is minimal; that is, the entropy of the mixture weights by which the bases are combined to explain the data point is low. A sparse code can now be obtained by imposing the *entropic prior* over the mixture weights. For a given distribution ***θ***, the entropic prior is defined as *P*(***θ***) ∝ *e* ^−*βℋ*(***θ***),^ where *ℋ*(***θ***) is the entropy. Imposition of this prior (with a positive *β*) on the mixture weights just means that we obtain solutions where mixture weights with low entropy are more likely to occur—a low entropy ensures that few entries of the vector are significant. Sparsity has been imposed in latent variable models by utilizing the entropic prior and has been shown to provide a better characterization of the data [17, 18, 23, 31]. Detailed derivation and estimation algorithms can be found in [17, 18]. Notice that priors can be imposed on any set of parameters during estimation.

**Figure 5:**
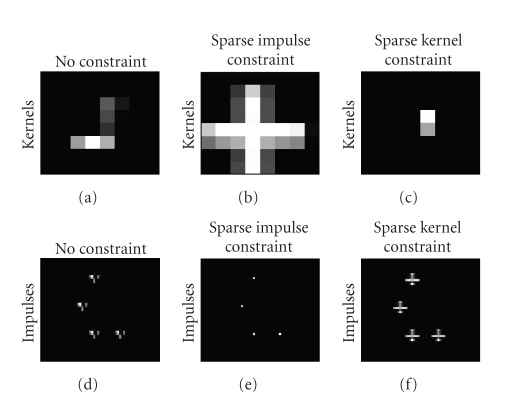
Example of the effect of the entropic prior on a set of kernel and impulse distributions. If no constraint is imposed, the information is evenly distributed among the two distributions (left column), if sparsity is imposed on the impulse distribution, most information lies in the kernel distribution (middle column), and vice verse if we request a sparse kernel distribution (right column).

Information theoretically, entropy is a measure of information content. One can consider the entropic prior as providing an explicit way to control the amount of “information content” desired on the components. We illustrate this idea using a simple shift-invariance case. Consider an image which is composed out of scattered plus sign characters. Upon analysis of that image, we would expect the kernel distribution to be a “+”, and the impulse distribution to be a set of delta functions placing it appropriately in space. However, using the entropic prior we can distribute the amount of information from the kernel distribution to the impulse distribution or vice-versa. We show the results from this analysis in Figure 5 in terms of three cases - where no entropic prior is used (left panels), where it is used to make the impulse sparse (mid panels), and where it is used to make the kernel sparse (right panels). In the left panels, information about the data is distributed both in the kernel (top) and in the impulse distribution (bottom). In the other two cases, we were able to concentrate all the information either in the kernel or in the impulse distribution by making use of the entropic prior.

Other prior distributions that have been used in various contexts include the Dirichlet [8, 32] and log-normal distributions [33] among others. The ability to utilize prior distributions during estimation provides a way to incorporate information known about the problem. More importantly, the probabilistic framework provides proven methods of statistical inference techniques that one can employ for parameter estimation. We point out that these extensions can work with all the generalizations that were presented in the previous sections.

### 4.4. Geometrical Interpretation

We also want to briefly point out that probabilistic models can sometimes provide insights that are helpful for an intuitive understanding of the workings of the model.

**Figure 6:**
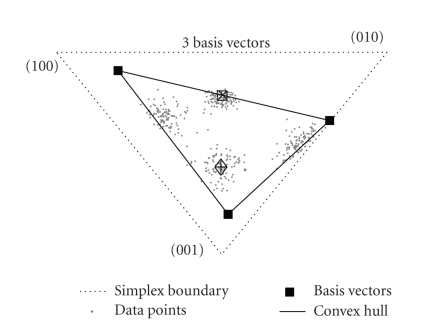
Illustration of the latent variable model. Panel shows 3-dimensional data distributions as points within the *Standard 2-Simplex* given by {(001), (010), (100)}. The model approximates data distributions as points lying within the convex hull formed by the components (basis vectors). Also shown are two data points (marked by + and ×) and their approximations by the model (resp., shown by *◊* and □).

Consider the asymmetric factorization case of the latent variable model as given by (6). Let us refer to the normalized columns of the data matrix **V** (obtained by scaling the entries of every column to sum to 1), ${\overset{¯}{v}}_{n}$, as *data distributions*. It can be shown that learning the model is equivalent to estimating parameters such that the model *P*(*x*_1_ | *x*_2_) for any data distribution ${\overset{¯}{v}}_{x_{2}}$ best approximates it. Notice that the data distributions ${\overset{¯}{v}}_{x_{2}}$, model approximations *P*(*x*_1_ | *x*_2_), and components *P*(*x*_1_ | *z*) are all *M*-dimensional vectors that sum to unity, and hence points in a (*M* − 1) simplex. The model expresses *P*(*x*_1_ | *x*_2_) as points within the convex hull formed by the components *P*(*x*_1_ | *z*). Since it is constrained to lie within this convex hull, *P*(*x*_1_ | *x*_2_) can model ${\overset{¯}{v}}_{x_{2}}$ accurately only if the latter also lies within the convex hull. Thus the objective of the model is to estimate *P*(*x*_1_ | *z*) as corners of a convex hull such that all the data distributions lie within. This is illustrated in Figure 6 for a toy dataset of 400 three-dimensional data distributions.

Not all probabilistic formulations provide such a clean geometric interpretation but in certain cases as outlined above, it can lead to interpretations that are intuitively helpful.

## 5. Discussion and Conclusions

In this paper, we presented a family of latent variable models and shown their utility in the analysis of nonnegative data. We show that the latent variable models decompositions are numerically identical to the NMF algorithm that optimizes a Kullback Leibler metric. Unlike previously reported results [34], the proof of equivalence requires no assumption about the distribution of the data, or indeed any assumption about the data besides nonnegativity. The algorithms presented in this paper primarily compute a probabilistic factorization of non-negative data that optimizes the KL distance between the factored approximation and the actual data. We argue that the use of this approach presents a much more straightforward way to make easily extensible models. (It is not clear that the approach can be extended to similarly derive factorizations that optimize other Bregman divergences such as the *L*2 metric—this is a topic for further investigation.)

To demonstrate this, we presented extensions that deal with tensorial data, shift invariances, and use priors on the estimation. The purpose of this paper is not to highlight the use of these approaches nor to present them thoroughly, but rather demonstrate a methodology which allows easier experimentation with nonnegative data analysis and opens up possibilities for more stringent and probabilistic modeling than before. A rich variety of real world applications and derivations of these and other models can be found in the references.
